# Parasitic infections in irritable bowel syndrome patients: evidence to propose a possible link, based on a case–control study in the south of Iran

**DOI:** 10.1186/s13104-020-05118-x

**Published:** 2020-06-01

**Authors:** Zohreh Shafiei, Farideh Esfandiari, Bahador Sarkari, Zahra Rezaei, Mohammad Reza Fatahi, Seyed Mohammad Kazem Hosseini Asl

**Affiliations:** 1 Department of Parasitology and Mycology, School of Medicine, Shiraz University of Medical Sciences, Shiraz, Iran; 2 Basic Sciences in Infectious Diseases Research Center, Shiraz University of Medical Sciences, Shiraz, Iran; 3 Professor Alborzi Clinical Microbiology Research Center, Shiraz University of Medical Sciences, Shiraz, Iran; 4 Department of Internal Medicine, Shiraz University of Medical Sciences, Shiraz, Iran

**Keywords:** IBS, Parasitic infection, *Blastocystis* spp., *Giardia lamblia*

## Abstract

**Objectives:**

The current study aimed to evaluate the prevalence of parasitic infections and their possible association with irritable bowel syndrome (IBS), through a case–control study. Stool samples were collected from patients with IBS and healthy subjects and were examined microscopically to detect intestinal parasites.

**Results:**

A total of 200 subjects were enrolled in the study including 100 patients with IBS and 100 healthy controls. The patients were selected based on the Rome III criteria. Of the 100 patients with IBS, 65 (65%) were female and 35 (35%) were male, with a mean age of 42.57 (± 4.07) years. Of these, 30 (30%) were infected with at least one intestinal parasite; the most common ones were *Blastocystis hominis* and *Giardia lamblia.* Of the control cases, 64 (64%) were female and 36 (36%) were male, with a mean age of 41.82 (± 11.75) years. Of these, 16 (16%) were infected with at least one intestinal parasite; the most common were *B. hominis* and *Endolimax*. There was a significant difference between the rate of parasitic infections between the patients with IBS and the control in particular, *B. hominis* and *G. lamblia.* The findings of the study support a possible link between parasitic infections and IBS.

## Introduction

Irritable bowel syndrome (IBS) is a group of diseases of the gastrointestinal tract that is associated with altered bowel movement and abdominal pain, without any known causes [1]. IBS has a profound negative impact on the lives of patients suffering from this syndrome and imposes significant economic and social costs on these patients [2, 3].

The prevalence of IBS is different in various regions of the world, based on the diagnostic criteria. In American and European countries, the prevalence of the disease is reported to be between 9 and 22 percent, respectively [4]. Countries such as India and Thailand [5, 6] have reported a low prevalence of 4.2% and 4.4%, respectively.

In Iran, Mahmoudi et al. [7] reported an IBS prevalence of 4.2% in a study, based on the Rome I criteria, among Tehran University students while Pourshams et al. [8] reported the prevalence of this syndrome as 4.7% in a population of students, based on Rome II criteria.

Numerous studies have been conducted to evaluate the association between parasitic, bacterial, and viral infections and IBS. *Blastocystis* and *Dientamoeba fragilis*, which are considered to be non-pathogenic parasites, have been linked to the etiology of IBS, in particular, the diarrhea-predominant IBS [9, 10]. While some studies have reported a significant association between the parasitic infections, especially *Blastocystis,* and IBS [9–11], in other studies no link has been found [12–14].

In a recent systematic review and meta-analysis focusing on the role of *Blastocystis* and *D. fragilis* in IBS, *Blastocystis* infection, but not *D. fragilis* infection was found to have a positive association with IBS [11]. Moreover, the meta-analysis reported subtypes 1 and 3 of *Blastocystis* as potential risk factors for IBS [11].

Considering the existing discrepancy in the association of parasitic infection and IBS, the current study was conducted to evaluate the prevalence of parasitic infections and their possible association with IBS, through a case–control study in Iran.

## Main text

### Methods

#### Study design

The IBS patients were selected from patients referred to internal clinics in university-affiliated hospitals in Shiraz, southern Iran. The inclusion criterion was having IBS, based on Rome III criteria, when examined by two gastroenterologists. The exclusion criteria were having celiac disease, gastrointestinal malignancies, or inflammatory bowel disease. The control subjects were recruited from a healthy population who referred to health centers for a routine check-up without having any complaint of gastrointestinal problems. A predesigned questionnaire was used to obtain sociodemographic, medical history, physical examination, and data related to IBS and parasitic infections. A fresh stool sample was collected from each participant for parasitological evaluation.

#### Parasitological evaluation of the fecal samples

The physical consistency of fresh fecal samples was classified as being formed or semi-formed. The fecal samples were grossly examined for the presence of blood, mucus, or adult helminthes. The samples were microscopically evaluated for intestinal parasites, using a wet preparation as well as a formalin/ethyl acetate concentration technique [15].

#### Statistical analysis

Statistical analysis of the data was performed, using SPSS (version 18; SPSS Inc., Chicago, IL, USA). The independent sample *t*-*test* was used to compare the means of different variables in two studied groups. Chi square, as well as Fisher’s exact tests, was used for comparing the discontinuous or categorical variables. P-value < 0.05 was considered statistically significant.

### Results

#### Features of IBS patients and their parasitic infection

A total of 200 subjects were enrolled in the study including 100 IBS patients and 100 healthy controls. Of the 100 patients with IBS, 65 (65%) were female and 35 (35%) were male, with a mean age of 42.57 ± 14.07 years (range 14-80 years). The mean duration of disease in the patient group was 4.33 (± 4.68) years with the lowest duration being 3 months and the longest 25 years.

Out of the 100 patients, 37 (37%) had an underlying disease, the most common of which were hypothyroidism (5%), hyperlipidemia (5%), coronary artery diseases (5%), and psychiatric disorders (4%).

Of the patients with IBS, 30 (30%) were infected with at least one intestinal parasite. The most common parasitic infections were *B. hominis* (15%) and *G. lamblia* (8%). Polyparasitism was found in 5 cases, including one (1%) with *G. lamblia* and *B. hominis*, two (2%) with *B. hominis* and *E. nana*, and 2 (2%) with *B. hominis* and *Entamoeba coli*.

With respect to daily stress and anxiety, 72 (72%) of the patients reported high levels of stress and anxiety. It should be noted that none of the patients with IBS had celiac disease, gastrointestinal malignancies, or inflammatory bowel disease, based on ultrasound, colonoscopy, and endoscopic examinations.

#### Features of control subjects and their parasitic infection

In the control group, 64 (64%) were female and 36 (36%) were male. The mean age of the control group was 41.82 (± 11.75) years, ranging from 17 to 75 years. The differences between the age and sex of the patients and controls were not significant when statistically analyzed.

In the control group, 16 (16%) cases were infected with at least one intestinal parasite; the most common were *B. hominis* and *E. nana.* In the control group in 86 (86%) cases, stool consistency was formed and in 14 cases (14%) it was semi-formed.

#### Comparison of the rate and diversity of parasitic infection in patients with IBS and controls

Analysis of data by independent sample *t*-*test* revealed a significant difference between the rate of parasitic infections in the patients with IBS and the control group (p = 0.019, df = 198, F = 23.64). There was also a statistically significant difference between patients with IBS and the control group regarding underlying diseases where the rate of these diseases was higher in patients with IBS. Furthermore, there was a statistically significant difference between the patients and the control group regarding the level of daily stress and anxiety, as well as the level of psychiatric disorders (p < 0.05). There was no statistically significant difference in the fecal specimen consistency between the patients and the control group.

With regard to the type of parasitic infection, there was a significant difference between the two groups concerning *G. lamblia* and *B. hominis* infections. In terms of polyparasitism, there was a significant statistical difference between the patients with IBS and the control group. Table 1 shows the demographic characteristics and differences between the control and Patients with IBS in the current study.

**Table 1:** Demographic features and differences between the control and the patients with IBS

Variable	Patients with IBS		Control		P-value
	Frequency	Percent	Frequency	Percent	
Sex					
Male	35	35	36	36	0.883
Female	65	65	64	64	
Age (year)					
≤ 20	6	6	3	3	0.683
21–30	15	15	17	17	
31–45	37	37	41	41	
46–60	31	31	36	36	
≥ 61	11	11	3	3	
Parasitic infection	30	30	16	16	0.019
Type of parasitic infection					
*Giardia lamblia*	8	8	0	0	0.004
*Blastocystis hominis*	15	15	6	6	0.038
*Endolimax nana*	5	5	6	6	0.758
*Chilomastix mesnili*	2	2	0	0	0.157
*Entamoeba coli*	2	2	4	4	0.410
*Egg of Enterobius vermicularis*	1	1	0	0	0.319
*Dientamoeba fragilis*	1	1	0	0	0.319
Living larvae	1	1	0	0	0.319
Mixed parasitic infections	5	5	0	0	0.024
Stool consistency					
Formed	84	84	86	86	0.694
Semi-formed	16	16	14	14	
Educational level					
Uneducated	11	11	0	0	0.001
High school or less	43	43	5	5	
High school diploma	21	21	11	11	
Undergraduate (AA or BA)	22	22	36	36	
Graduate (MSc or Ph.D.)	3	3	48	48	
Occupation					
Unemployed	2	2	1	1	0.001
Housewife	53	53	21	21	
Self-employed	18	18	8	8	
Student	5	5	13	13	
Clerk	22	22	57	57	
Level of stress and anxiety					
High	72	72	26	26	0.001
Low	28	28	74	74	
Psychiatric disorders	4	4	0	0	0.044

### Discussion

The present study investigated the prevalence of intestinal parasites in patients with IBS and healthy controls. Of the 100 patients studied, 30 (30%) were infected with at least one intestinal parasite and *G. lamblia* and *Blastocystis spp.* were the most common. Differences in the rate of parasitic infection, in particular *B. hominis* and *G. lamblia*, between the patients with IBS and controls were statistically significant.

*Blastocystis* spp. is a common intestinal protozoan and its role as a parasitic pathogen is still controversial. The contribution of *Blastocystis* to IBS has been proposed in several studies [9–11]. The findings of the current study are consistent with previous studies, in that a higher rate of parasitic infection in patients with IBS was presented, in comparison with the healthy controls [9–11]. In Jimenez-Gonzalez et al. study [9], a significant association between *Blastocystis* infection and IBS was reported in the Mexican patient population, where the rate of infection with this parasite was higher in patients with IBS than in the control group.

In concordance with our findings, other reports from Europe, Mexico, India, the Middle East, and Pakistan, proposed for the role of *Blastocystis* in the pathogenicity of IBS [11, 16, 17]. Moreover, a systematic review and meta-analysis in Iran supported the existence of a positive association between *Blastocystis* infection and IBS [11]. The yield of infection in patients with IBS has been reported to be higher than that of healthy counterparts, where patients with IBS had significantly more *B. hominis* per microscopic field [18].

On the other hand, several studies have not found a positive association between *Blastocystis* infection and IBS [12–14, 19]. Krogsgaard et al. [20] showed that both *D. fragilis* and *Blastocystis* spp. parasites are more prevalent in healthy subjects than in patients with IBS. Yakoob et al. [10] evaluated the rate of *B. hominis* and *D. fragilis* infections in IBS-diarrhea patients and controls. Although the rate of both infections was higher in patients with IBS, the author concluded that a prospective study with a larger population was needed to support any association between IBS and these parasitic infections. In another study by Khademvatan et al. [12] in Khuzestan, south of Iran, 122 patients with IBS and 122 healthy individuals were evaluated for *Blastocystis* infection where no statistically significant difference was found between the two groups. In another study in Iran, Beiromvand et al. [21] demonstrated that *Blastocystis* spp. was more prevalent in the control group in comparison with the patients with IBS. In a study in Thailand, [13] no significant difference was found in the incidence of *Blastocystis* in patients with IBS and healthy controls. In Morgan et al. [22] study no association was found between IBS and parasitic infections in a developing nation environment, Leon Municipality, where the rate of parasitic infection is substantial.

In the current study, *G. lamblia* was more prevalent in stool samples of patients with IBS than in the controls. This is in agreement with previous studies that reported a higher rate of *G. lamblia* in patients with IBS [16, 23].

The association between the genotypes of *B. hominis* with IBS is controversial. In a study by Yakoob et al. [17] in Karachi, Pakistan, type 1 of *Blastocystis* was predominant in the patients with IBS whereas type 3 was more common in the control group. Also researchers from Egypt reported genotype I as the dominant genotype of *B. hominis* in patients with IBS [24]. Such association was not found in the study of Khademvatan et al., [12] in Iran. In our study, the genotypes of *B. hominis* were not determined.

### Conclusion

In this case–control study, concerning the potential role of parasitic infection in IBS status, a significant difference in the prevalence of *Blastosystis spp* and *G. lamblia* was seen in patients with IBS in comparison with the control group. The findings are in line with those researchers who reported a link between parasitic infections and IBS. Whether this is a causative, risk factor or an association between parasitic infection and IBS deserve further studies. Results of the present study also support an association between psychiatric disorders and IBS. Further studies with a larger sample size are needed to clarify the role of parasitic infections in the pathogenesis of IBS.

## Limitations

There are several limitations to this study. First, the sample size of the study is relatively small. Second, the genotypes of *Blastocystis* have not been assessed to investigate the possible association of the *Blastocystis* subtypes and IBS. Also, the literacy levels of the two groups were somewhat different, which may, to some extent, have affected the rate of parasite infection in the two studied groups.

## Data Availability

Any further requested information regarding the experimental and data analysis during the current study is available from the corresponding author on request.

## Abbreviation

IBS: Irritable bowel syndrome

## References

[1] Locke GR, Olden K, Peterson W, Quigley E, Schoenfeld P, Schuster M, Talley N (2002). Systematic review on the management of irritable bowel syndrome in North America. Am J Gastroenterol.

[2] Chang L (2004). Review article: epidemiology and quality of life in functional gastrointestinal disorders. Aliment Pharmacol Ther.

[3] Hulisz D (2004). The burden of illness of irritable bowel syndrome: current challenges and hope for the future. J Manag Care Pharm..

[4] Cremonini F, Talley NJ (2005). Irritable bowel syndrome: epidemiology, natural history, health care seeking and emerging risk factors. Gastroenterol Clin North Am.

[5] Danivat D, Tankeyoon M, Sriratanaban A (1988). Prevalence of irritable bowel syndrome in a non-Western population. Br Med J.

[6] Ghoshal UC, Abraham P, Bhatt C, Choudhuri G, Bhatia SJ, Shenoy KT, Banka NH, Bose K, Bohidar NP, Chakravartty K (2008). Epidemiological and clinical profile of irritable bowel syndrome in India: report of the Indian Society of Gastroenterology Task Force. Indian J Gastroenterol.

[7] Mahmudi S, Pourshams A, Akbari M, Malekzadeh R (2012). The prevalence of irritable bowel syndrome and gastroesophageal reflux disease among Tehran University students. Govaresh..

[8] Pourshams A, Zendehdel N, Semnani M, Semnani U (2012). Irritable bowel syndrome and psychiatric disorders Amang University Freshmen. Govaresh..

[9] Jimenez-Gonzalez DE, Martinez-Flores WA, Reyes-Gordillo J, Ramirez-Miranda ME, Arroyo-Escalante S, Romero-Valdovinos M, Stark D, Souza-Saldivar V, Martinez-Hernandez F, Flisser A (2012). Blastocystis infection is associated with irritable bowel syndrome in a Mexican patient population. Parasitol Res.

[10] Yakoob J, Jafri W, Beg MA, Abbas Z, Naz S, Islam M, Khan R (2010). Blastocystis hominis and Dientamoeba fragilis in patients fulfilling irritable bowel syndrome criteria. Parasitol Res.

[11] Rostami A, Riahi SM, Haghighi A, Saber V, Armon B, Seyyedtabaei SJ (2017). The role of *Blastocystis* sp. and *Dientamoeba fragilis* in irritable bowel syndrome: a systematic review and meta-analysis. Parasitol Res.

[12] Khademvatan S, Masjedizadeh R, Rahim F, Mahbodfar H, Salehi R, Yousefi-Razin E, Foroutan M (2017). Blastocystis and irritable bowel syndrome: frequency and subtypes from Iranian patients. Parasitol Int.

[13] Surangsrirat S, Thamrongwittawatpong L, Piyaniran W, Naaglor T, Khoprasert C, Taamasri P, Mungthin M, Leelayoova S (2010). Assessment of the association between Blastocystis infection and irritable bowel syndrome. J Med Assoc Thai.

[14] Tungtrongchitr A, Manatsathit S, Kositchaiwat C, Ongrotchanakun J, Munkong N, Chinabutr P, Leelakusolvong S, Chaicumpa W (2004). Blastocystis hominis infection in irritable bowel syndrome patients. Southeast Asian J Trop Med Public Health.

[15] Sarkari B, Hosseini G, Motazedian MH, Fararouei M, Moshfe A (2016). Prevalence and risk factors of intestinal protozoan infections: a population-based study in rural areas of Boyer-Ahmad district, Southwestern Iran. BMC Infect Dis.

[16] Jadallah KA, Nimri LF, Ghanem RA (2017). Protozoan parasites in irritable bowel syndrome: a case-control study. World J Gastrointest Pharmacol Ther..

[17] Yakoob J, Jafri W, Beg MA, Abbas Z, Naz S, Islam M, Khan R (2010). Irritable bowel syndrome: is it associated with genotypes of Blastocystis hominis. Parasitol Res.

[18] Giacometti A, Cirioni O, Fiorentini A, Fortuna M, Scalise G (1999). Irritable bowel syndrome in patients with *Blastocystis hominis* infection. Eur J Clin Microbiol Infect Dis.

[19] Krogsgaard LR, Andersen LO, Johannesen TB, Engsbro AL, Stensvold CR, Nielsen HV, Bytzer P (2018). Characteristics of the bacterial microbiome in association with common intestinal parasites in irritable bowel syndrome. Clin Transl Gastroenterol..

[20] Krogsgaard LR, Engsbro AL, Stensvold CR, Nielsen HV, Bytzer P (2015). The prevalence of intestinal parasites is not greater among individuals with irritable bowel syndrome: a population-based case-control study. Clin Gastroenterol Hepatol.

[21] Beiromvand M, Hashemi SJ, Arjmand R, Sadjadei N, Hardanipasand L (2017). Comparative prevalence of blastocystis in patients with the irritable bowel syndrome and healthy individuals: a case control study. Jundishapur J Microbiol..

[22] Morgan DR, Benshoff M, Caceres M, Becker-Dreps S, Cortes L, Martin CF, Schmulson M, Pena R (2012). Irritable bowel syndrome and gastrointestinal parasite infection in a developing nation environment. Gastroenterol Res Pract..

[23] Hanevik K, Dizdar V, Langeland N, Hausken T (2009). Development of functional gastrointestinal disorders after Giardia lambliainfection. BMC Gastroenterol..

[24] Fouad SA, Basyoni MM, Fahmy RA, Kobaisi MH (2011). The pathogenic role of different Blastocystis hominis genotypes isolated from patients with irritable bowel syndrome. Arab J Gastroenterol.

